# Supplementary material for: Impaired FADD/BID signaling mediates cross-resistance to immunotherapy in Multiple Myeloma
Source: Commun Biol. 2023 Dec 21;6:1299. doi: 10.1038/s42003-023-05683-4 (PMC10739907; doi:10.1038/s42003-023-05683-4)
Supplement: Supplementary file 2 — Supplementary Information [file 42003_2023_5683_MOESM2_ESM.pdf]

Figure S1

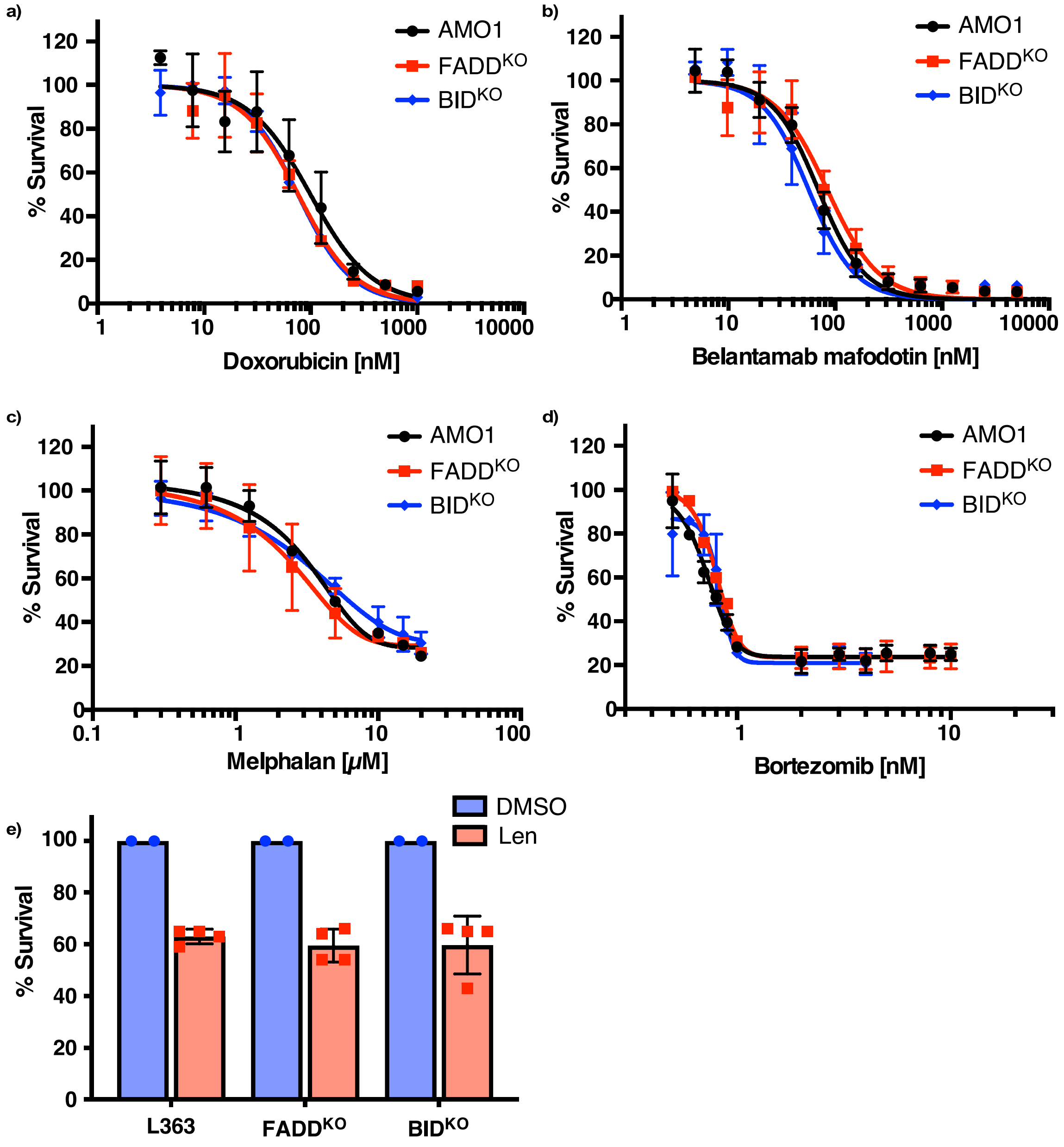

Figure S1) No affect was observed on activity of conventional antimyeloma drugs. AMO1 cells were treated with doxorubicin (A), belantamab mafodotin (B), melphalan (C) and bortezomib (D) for 72 hours, L363 cells with10µM lenalidomide (Len) for 120 hours (E) and subjected to alamarblue assay.

Figure S2

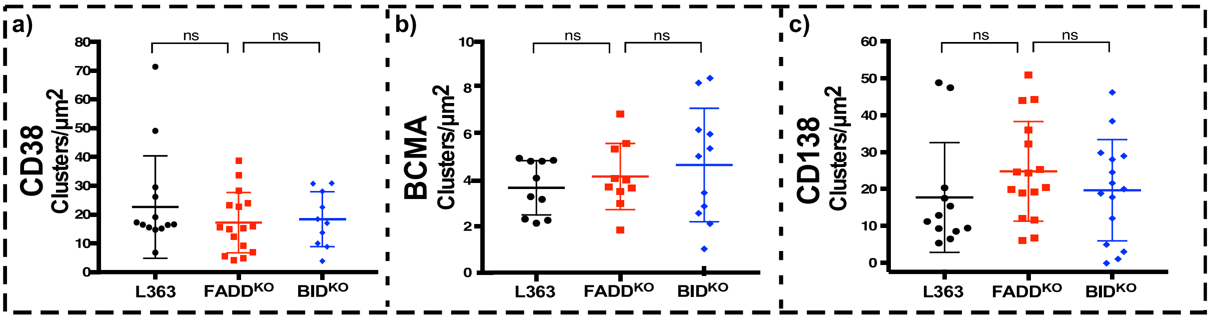

Figure S2) No significant differences in expression level of CD38, BCMA and CD138 target antigen were observed in FADD^{KO} and BID^{KO} when compared to WT L363 cells observed with *d*STORM.

Figure S3

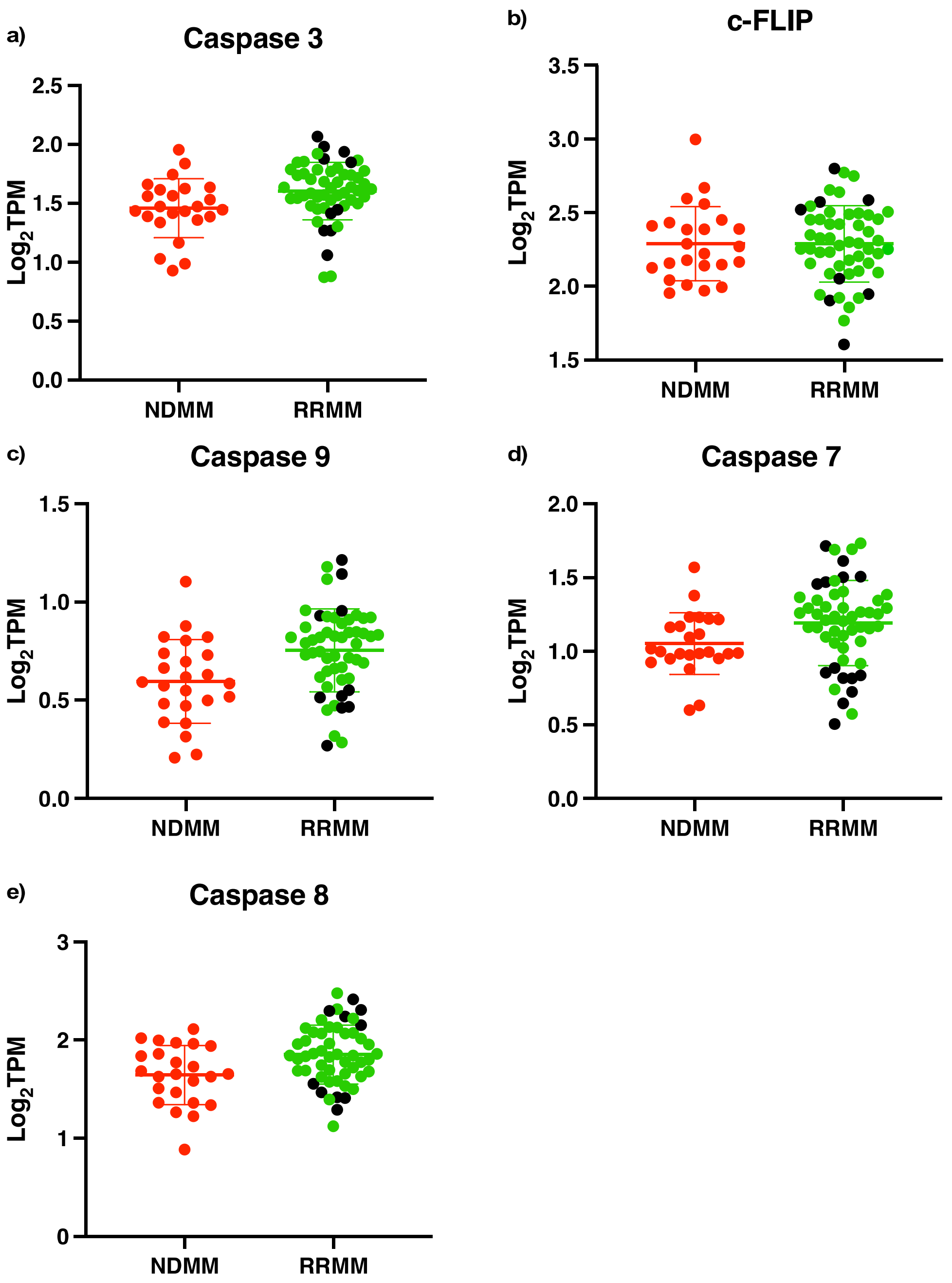

Figure S3) Gene expression data of Caspase 3 (A) c-FLIP (B) Caspase 9 (C) Caspase 7 (D) and Caspase 8 (E) in healthy donors (HD), NDMM and RRMM patients. Gene expression was determined by bulk RNA seq and normalized as log_2TPM and quantified by qPCR and normalized as $2^{\Delta CT}$.

Figure S4

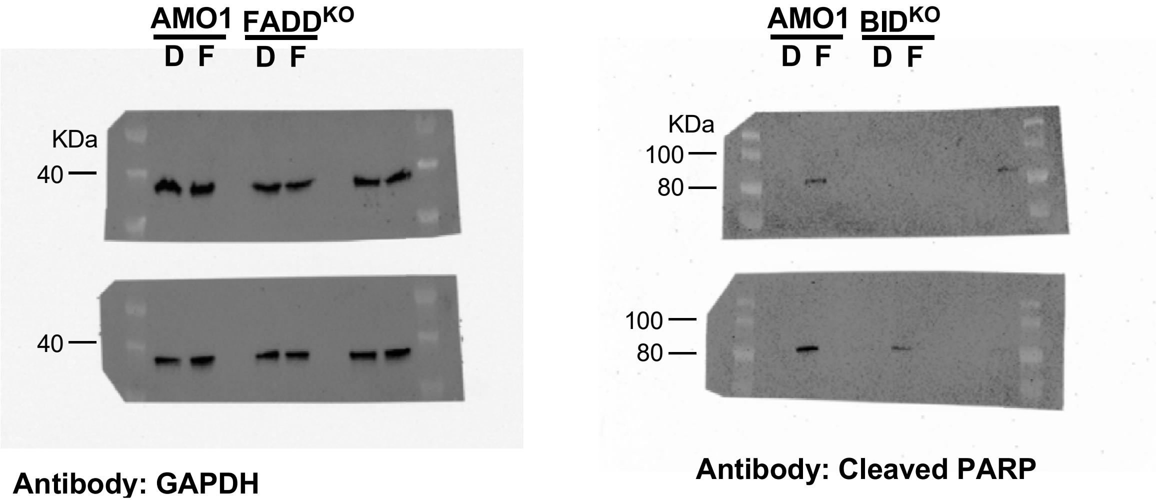

Figure S4) Full length blots stained for Cleaved PARP and GAPDH.

Supplementary Table 1: *FADD* and *BID* expression in RRMM patients resistant to daratumumab

No	Gene	Log_2TPM	Subtype	Age at diagnosis	Cytogenetics	Line of therapy	Drug exposure			
							PI	IMiD	mAb	SCT
**1**	FADD	0.922098874	IgG Kappa	59	Standard-risk	5	BTZ, CFZ	LEN, POM	DARA, ELO	AUTO
**2**	FADD	1.46456372	Kappa LC	46	High-risk	6	BTZ, CFZ	LEN, POM	DARA	AUTO
**3**	FADD	1.520306577	IgA Lambda	68	High-risk	4	BTZ, CFZ	LEN, POM	DARA	AUTO
**4**	FADD	1.775914424	Kappa LC	72	High-risk	3	BTZ, CFZ	POM	DARA	AUTO
**5**	FADD	1.870991971	Lambda LC	55	Standard-risk	9	BTZ, CFZ	LEN, POM	DARA, ELO	AUTO
**6**	FADD	1.990918566	IgG Kappa	51	Standard-risk	5	CFZ	LEN	DARA	AUTO
**7**	FADD	1.999520224	IgA Lambda	57	High-risk	2	BTZ	LEN	DARA	AUTO
**8**	FADD	3.362077074	IgG Kappa	58	High-risk	3	BTZ, CFZ	LEN	DARA	AUTO
**9**	FADD	3.643278996	IgA Kappa	53	High-risk	4	BTZ	LEN	DARA	AUTO
**10**	FADD	5.005292186	Kappa LC	56	High-risk	8	BTZ, CFZ	LEN, POM	DARA, ELO	AUTO
**11**	BID	2.27402604	IgG Kappa	60	High-risk	4	BTZ, CFZ	LEN	DARA	AUTO

**12**	BID	2.34807091	IgM Kappa	55	High-risk	4	BTZ, CFZ	LEN, POM	DARA, ELO	AUTO
**13**	BID	2.44581938	IgA Kappa	64	High-risk	3	BTZ, CFZ	LEN, POM	DARA	AUTO
**14**	BID	2.7016289	Kappa LC	73	High-risk	4	BTZ, CFZ	LEN, POM	DARA	AUTO
**15**	BID	2.74444315	IgG Kappa	59	Standard-risk	5	BTZ, CFZ	LEN, POM	DARA, ELO	AUTO
**16**	BID	2.75219838	IgG Kappa	72	High-risk	4	BTZ	LEN, POM	DARA	AUTO
**17**	BID	2.77720086	IgA Kappa	52	High-risk	3	BTZ, CFZ	LEN, POM	DARA	-
**18**	BID	2.82396942	Kappa LC	46	High-risk	6	BTZ, CFZ	LEN, POM	DARA	AUTO
**19**	BID	2.87298828	IgG Kappa	55	Standard-risk	7	BTZ, CFZ	LEN, POM	DARA, ELO	AUTO
**20**	BID	4.46726644	Kappa LC	51	High-risk	2	BTZ	LEN	DARA	-
**21**	BID	4.70487196	IgG Lambda	59	High-risk	2	BTZ	LEN	DARA	-
**22**	BID	5.09396575	IgG Lambda	65	High-risk	9	BTZ, CFZ	LEN, POM	DARA	AUTO

AUTO - autologous stem cell transplant, BTZ - bortezomib, CFZ - carfilzomib, DARA - daratumumab, ELO - elotuzumab, LC - light chain, LEN - lenalidomide, POM- pmalidomide, RRMM - relapsed/refractory multiple myeloma, SCT - stem cell transplant

Supplementary Table 2: CRISPR gRNA sequences

Name	Sequence 5‘ - 3‘
**FADD- CRISPR-Oligo-F**	TGACGTTAAATGCTGCACACGTTTT
**FADD- CRISPR-Oligo-R**	GTGTGCAGCATTTAACGTCACGGTG
**BID- CRISPR-Oligo-F**	ACGTGAGGAGCTTAGCCAGAGTTTT
**BID- CRISPR-Oligo-R**	ACGTGAGGAGCTTAGCCAGAGTTTT
