# Supplementary material for: Impaired FADD/BID signaling mediates cross-resistance to immunotherapy in Multiple Myeloma
Source: Commun Biol. 2023 Dec 21;6:1299. doi: 10.1038/s42003-023-05683-4 (PMC10739907; doi:10.1038/s42003-023-05683-4)
Supplement: Supplementary file 5 — Reporting Summary [file 42003_2023_5683_MOESM5_ESM.pdf]

nature portfolio

Corresponding author(s): Martin Kortüm

Last updated by author(s): 27.11.2023

Reporting Summary

Nature Portfolio wishes to improve the reproducibility of the work that we publish. This form provides structure for consistency and transparency in reporting. For further information on Nature Portfolio policies, see our Editorial Policies and the Editorial Policy Checklist.

Statistics

For all statistical analyses, confirm that the following items are present in the figure legend, table legend, main text, or Methods section.

n/a	Confirmed	
☐	☒	The exact sample size (*n*) for each experimental group/condition, given as a discrete number and unit of measurement
☒	☐	A statement on whether measurements were taken from distinct samples or whether the same sample was measured repeatedly
☐	☒	The statistical test(s) used AND whether they are one- or two-sided<br>*Only common tests should be described solely by name; describe more complex techniques in the Methods section.*
☒	☐	A description of all covariates tested
☒	☐	A description of any assumptions or corrections, such as tests of normality and adjustment for multiple comparisons
☐	☒	A full description of the statistical parameters including central tendency (e.g. means) or other basic estimates (e.g. regression coefficient) AND variation (e.g. standard deviation) or associated estimates of uncertainty (e.g. confidence intervals)
☐	☒	For null hypothesis testing, the test statistic (e.g. *F*, *t*, *r*) with confidence intervals, effect sizes, degrees of freedom and *P* value noted<br>*Give P values as exact values whenever suitable.*
☒	☐	For Bayesian analysis, information on the choice of priors and Markov chain Monte Carlo settings
☒	☐	For hierarchical and complex designs, identification of the appropriate level for tests and full reporting of outcomes
☒	☐	Estimates of effect sizes (e.g. Cohen's *d*, Pearson's *r*), indicating how they were calculated

Our web collection on statistics for biologists contains articles on many of the points above.

Software and code

Policy information about availability of computer code

Data collection	No Code was used.
Data analysis	Prism 7. Flowjo 9.

For manuscripts utilizing custom algorithms or software that are central to the research but not yet described in published literature, software must be made available to editors and reviewers. We strongly encourage code deposition in a community repository (e.g. GitHub). See the Nature Portfolio guidelines for submitting code & software for further information.

Data

Policy information about availability of data

All manuscripts must include a data availability statement. This statement should provide the following information, where applicable:

- Accession codes, unique identifiers, or web links for publicly available datasets
- A description of any restrictions on data availability
- For clinical datasets or third party data, please ensure that the statement adheres to our policy

Guide RNA sequences and Log2TPM values are provided as supplementary tables. Other data is provided as supplementary data file.

Research involving human participants, their data, or biological material

Policy information about studies with human participants or human data. See also policy information about sex, gender (identity/presentation), and sexual orientation and race, ethnicity and racism.

Reporting on sex and gender	Sex and gender based analysis were not relevant for the study
Reporting on race, ethnicity, or other socially relevant groupings	Race, ethnicity or social relevant grouping were not relevant for the study.
Population characteristics	N/A
Recruitment	No participants were recruited in the study. Data available from routine diagnostics was used after informed consent.
Ethics oversight	N/A

Note that full information on the approval of the study protocol must also be provided in the manuscript.

Field-specific reporting

Please select the one below that is the best fit for your research. If you are not sure, read the appropriate sections before making your selection.

☒ Life sciences ☐ Behavioural & social sciences ☐ Ecological, evolutionary & environmental sciences

For a reference copy of the document with all sections, see nature.com/documents/nr-reporting-summary-flat.pdf

Life sciences study design

All studies must disclose on these points even when the disclosure is negative.

Sample size	No Statistical tests were used to determine correct sample size. Routine data from all the patients undergoing Daratumumab treatment at University Hospital Würzburg were included in the study.
Data exclusions	No data excluded.
Replication	All the invitro data was reproducible and each experiment was perfomed atleast 3 times.
Randomization	N/A
Blinding	Blinding was not applicable, study used data from routine diagnostics

Reporting for specific materials, systems and methods

We require information from authors about some types of materials, experimental systems and methods used in many studies. Here, indicate whether each material, system or method listed is relevant to your study. If you are not sure if a list item applies to your research, read the appropriate section before selecting a response.

Materials & experimental systems

n/a	Involved in the study	
☐	☒	Antibodies
☐	☒	Eukaryotic cell lines
☒	☐	Palaeontology and archaeology
☒	☐	Animals and other organisms
☐	☒	Clinical data
☒	☐	Dual use research of concern
☒	☐	Plants

Methods

n/a	Involved in the study	
☒	☐	ChIP-seq
☐	☒	Flow cytometry
☒	☐	MRI-based neuroimaging

Antibodies

Antibodies used	Anti-Fas Antibody (human, activating), clone CH11, 05-201 Merck, Cleaved PARP (Asp214) (D64E10) XP® Rabbit mAb #5625 (cell signaling)

Validation	Each antibody has been cited multiple times in literature with publications mentioned on website.

Eukaryotic cell lines

Policy information about cell lines and Sex and Gender in Research

Cell line source(s)	DSMZ (German Colleciton of Microorganisms and Cell cultures GmBH)
Authentication	Cell lines were purchased from DSMZ
Mycoplasma contamination	Cells cultures were tested regularly for mycoplasma contamination
Commonly misidentified lines (See ICLAC register)	N/A

Clinical data

Policy information about clinical studies

All manuscripts should comply with the ICMJE guidelines for publication of clinical research and a completed CONSORT checklist must be included with all submissions.

Clinical trial registration	N/A
Study protocol	N/A
Data collection	N/A
Outcomes	N/A

Flow Cytometry

Plots

Confirm that:

- [x] The axis labels state the marker and fluorochrome used (e.g. CD4-FITC).
- [x] The axis scales are clearly visible. Include numbers along axes only for bottom left plot of group (a 'group' is an analysis of identical markers).
- [x] All plots are contour plots with outliers or pseudocolor plots.
- [x] A numerical value for number of cells or percentage (with statistics) is provided.

Methodology

Sample preparation	Cells were washed with PBS and resuspended in Annexin-V binding buffer (Immunostep), followed by incubation with 2µl Annexin DY 634 at RT for 15 minutes. After washing once with PBS, cells were resuspended in 200µl of buffer containing 2µl PI.
Instrument	cytoflex (Beckman Coulter)
Software	Flowjo 9.0
Cell population abundance	N/A. Whole cell population without sorting was used for analysis
Gating strategy	The only gating strategy employed in the study was defined based on size/granularity of cell lines on FSC-SSC to distinguish cells from debris.

- [ ] Tick this box to confirm that a figure exemplifying the gating strategy is provided in the Supplementary Information.
